# Effect of Incisal Height on Fracture Resistance of Lithium Disilicate and Hybrid Ceramic Laminate Veneers

**DOI:** 10.1155/ijod/8923600

**Published:** 2025-09-29

**Authors:** Negin Yaghoobi, Majid Sahebi, Farzaneh Farid

**Affiliations:** School of Dentistry, Tehran University of Medical Sciences, Tehran, Iran

**Keywords:** dental laminate veneers, fracture resistance, incisal height, IPS e.max CAD, VITA Enamic

## Abstract

**Objectives:**

This in vitro study evaluated the effect of the incisal height and the type of restorative material on the fracture resistance of labial laminate veneers in teeth with severe loss of incisal edge.

**Methods:**

In total, thirty-two human maxillary central incisors were randomly allocated into two groups (*n* = 16). In one group the incisal edge was reduced 2 mm and in the other group 3.5 mm. In each group half of the laminates were made of lithium disilicate (IPS e.max CAD) and the other half of a polymer-infiltrated ceramic network (PICN; VITA Enamic). After cementation of laminates with a resin cement (Choice 2), all specimens were subjected to thermal cycling (5−55°C, dwell time 30 s, 5000 cycles) and cyclic loading (30 N, 1.3 Hz, 500,000 cycles). Next, specimens were loaded to failure in a universal testing machine, with a crosshead speed of 1 mm/min. The data were analyzed using two-way analysis of variance (ANOVA) and *T*-test (*α* = 0.05).

**Results:**

Mean load to failure of laminates with 2 mm and 3.5 mm incisal height was respectively, 620.11 N and 901.81 N, in e.max CAD; and 466.43 N and 460.14 N in the VITA Enamic group. Only in e.max CAD laminates was the difference between two subgroups was significant(*p*=0.025).

**Conclusions:**

According to the results of this study, increasing the incisal height of e.max CAD laminates increases their load to fracture. However, it has no significant effect on VITA Enamic laminates.

**Clinical Significance:**

For anterior teeth that have lost more than 2 mm of incisal height, laminates made of lithium disilicate can be considered as a reliable option.

## 1. Introduction

In human dentition anterior teeth play important tasks, including but not limited to cooperating in mastication, speech and guiding the mandible in eccentric movements. Although, their most noticeable function is in the humans' social life through an appealing smile [1, 2].

Different procedures and materials are proposed for restoring the form and function of damaged anterior teeth [3]. Some of these techniques, like full veneer crowns require heavy preparation of tooth structure [4]. To address unnecessary reduction of sound dental tissues, laminate veneers were introduced to dentistry [4, 5]. These restorations conservatively restore discolored, malformed, worn, or traumatized anterior teeth. Although they are relatively thin, their strong bond to enamel enables them to survive in oral environment [5–7].

Initially, laminate veneers were proposed as no-prep restorations. But later, a 0.3 mm to 1.0 mm reduction of the facial surface was recommended in order to remove the aprismatic outer layer of enamel and improve bond strength and to avoid bulky gingival contours [8, 9]. Similarly, the preparation of the incisal area evolved from almost no or minimal prep (window and feather edge designs) to the reduction of the incisal edge with or without lingual extension (palatal chamfer and butt joint designs). The type of incisal preparation depends on the clinical situation, esthetic demands and the kind of restorative material [10, 11].

In metal–ceramic crowns, 2 mm thickness of porcelain is an accepted limit, based on its cohesive strength. Manufacturers of dental ceramics also claim that laminate veneers can have 2 mm incisal height without compromising their strength [12, 13]. However, in everyday dentistry, it is not uncommon to see otherwise intact teeth with more than 2 mm loss of the incisal height due to the trauma, attrition, or abrasion [14]. Because of the extension of destruction, most of the time these cases are restored with full crowns [15]. Of course, a more conservative still durable treatment is more beneficial to the remaining tooth structure and the whole dentition [16]. Laminate veneers could be a conservative alternative in these situations; however, their longevity with increased incisal height is questionable [13, 17].

Factors affecting the success or failure of ceramic laminate veneers can be summarized into four categories: factors related to tooth substrate, such as the quality and quantity of remaining enamel or dentin and the preparation design; factors associated with the restorative material, including its mechanical properties, thickness, flexural strength, and fracture toughness; and factors correlated to the cementing medium, like its type, thickness, and bond strength to the restoration and tooth structure. Of course, the direction of masticatory load and force-intensifying factors should not be overlooked [18–21].

There are a few studies that have investigated the effect of incisal height on the fracture strength of laminate veneers [13, 18]. These studies are almost inclusively on leucite-based glass–ceramics [17, 22]. Nevertheless, in recent years stronger glass ceramics have been introduced to dentistry [23, 24]. Lithium disilicates are durable highly esthetic glass–ceramics with a flexural strength of 360–500 MPa [25]. They are indicated for partial or full coverage restorations [10, 12]. The first launched lithium disilicate was IPS Empress 2, followed by IPS e.max Press. The former was composed of 65 vol% lithium disilicate in a glass matrix [26]. The latter is stronger because it is manufactured by bulk casting rather than via a powder stage and has tightly packed elongated disilicate crystals. The crystals participate in the toughening of the ceramic by a crack-bridging mechanism, and along with the thermal expansion mismatch between them and the glass matrix that causes tangential, compressive stress around the crystals, increase the strength of the ceramic [26]. The latest product of this line is IPS e.max CAD, of claimed 500 MPa flexural strength with further improvement in the translucency as a result of the development of its crystalline volume and refractive index [24, 25].

On the other hand, polymer-infiltrated ceramic network (PICN) is a new line of hybrid ceramics developed to address the brittleness of ceramics and render more similarity to the properties of natural tooth structure. These materials combine the characteristics of ceramics and composites. However, because of high ceramic content, they have fewer disadvantages than composites in marginal adaptation, color match, and anatomic form [27]. VITA Enamic is one of these materials developed for CAD/CAM technology. Based on the literature, its skeleton (86% by weight) is formed by a leucite-based ceramic of feldspar origin (58%–63%), aluminum oxide (20%–23%), and a minor crystalline zirconia phase, perhaps as a strengthening component [27]. This porous network is conditioned by a coupling agent, and then a UDMA resin (14% by weight) interpenetrates into it by capillary action. According to the manufacturer, its flexural strength is 150–160 MPa and is recommended for anterior and posterior single crowns, inlays, onlays, occlusal, and laminate veneers. Compared to silicate ceramics, it is possible to mill Enamic restorations with thinner walls. That is particularly suitable for minimally invasive restorations. Its abrasion is like enamel and protects the antagonist tooth because of its fine ceramic network structure. It has higher elasticity than traditional dental ceramics since the acrylate polymer network provides flexibility. Its dual network structure has a crack-stop function.

Manufacturers are clear about the minimum incisal preparation for different restorations and restorative materials. But there is no direct suggestion regarding the maximum reliable thickness of the material, especially in the incisal part of laminate veneers. Since there are situations in which a large portion of the incisal area is lost, from a clinical point of view, it is valuable to investigate whether lithium disilicate and PICN are strong enough for laminate veneers restoration of these teeth. The aim of this study was to evaluate the effect of incisal height on the fracture resistance of labial laminate veneer made out of IPS e.max CAD and VITA Enamic. The null hypothesis was the load to fracture would not change by increasing the incisal height.

## 2. Materials and Methods

This study was approved by the Ethical Committee at the Tehran University of Medical Sciences (IR.TUMS.DENTISTRY.REC.1399.080). Assuming *α* = 0.05, *β* = 0.1%, and 85% study power, the suggested sample size was 32, eight per each group.

Therefore, 32 human maxillary central incisors with approximately 10.5 ± 0.5 mm crown height and 8 ± 0.5 mm crown width were selected. The teeth were unrestored, without caries and cracks, and recently extracted for periodontal or other therapeutic reasons. They were cleaned by scaling and kept in saline solution until the study began, which was in less than 1 month.

### 2.1. Tooth Preparation

**Figure 1:**
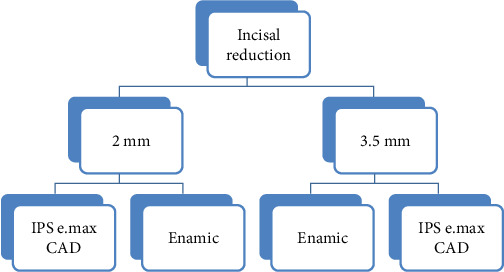
Diagram of groups.

To prepare the teeth for laminate veneers, all labial surfaces were reduced 0.5 mm at the incisal two-thirds and 0.3 mm at the cervical one-third. The shallow chamfer cervical finish line was established 1 mm above the cement–enamel junction. The reduction extended to the middle of each proximal surface. For the incisal edge preparation, the teeth were assigned to two groups. In one group the incisal edge was reduced 2 mm, and in the other group 3.5 mm. In all the teeth, the preparation was extended 1.5 mm to the lingual with a chamfer finish line (Figure 1).

### 2.2. Fabrication of Veneers

**Figure 2:**
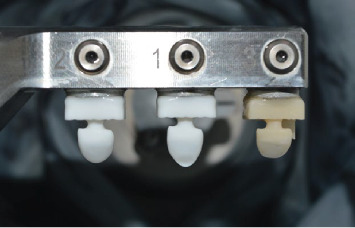
Fabrication of laminates.

After completion of the tooth preparation, all the teeth were covered with opaque powder (Alldent, Zahnzentrum, Stuttgart, Germany) and scanned (Ceramill Map 400 Scanner, Amann Girrbach, Germany). The data was transferred to EXO CAD software, and regardless of the length of incisal reduction, laminates with the same height were designed for all the specimens. In this way, the incisal height in half of the laminates was 2 mm and in the other half 3.5 mm. The laminates were milled from IPS e.max CAD (LD2 and LD3.5 groups) or VITA Enamic (PICN2 or PICN3.5 groups) (Figure 2).

LD laminates were milled from partially crystalized HT blocks (IPS-e.max CAD, Ivoclar Vivadent AG, Schaan, Liechtenstein) and were fully crystallized in a ceramic furnace according to the manufacturer's instructions for use. After that, a glaze layer was applied on laminates and fired at 765°C.

In PICN groups, the veneers were milled from VITA Enamic blocks (VITA Enamic, VITA Zahnfabrik H. Rauter GmbH and Co. Germany) without any further sintering or crystallization according to the manufacturer's instruction for use. They were just finished and polished.

All the laminate veneers that had marginal discrepancy or those without a passive fit on the prepared tooth were excluded from the study, and the process of scanning and fabrication was repeated.

Then the internal surface of the laminates was air-abraded with 50 µm aluminum oxide particles (Cojet; 3M ESPE) at 2.8 bar pressure and 10 mm distance, cleaned by pumice slurry, rinsed and dried [28].

### 2.3. Cementation

For cementation, the fitting surface of all laminates was etched with 9.5% hydrofluoric acid (BISCOSchaumburg, USA). After 20 s for LD and 60 s for PICN groups, the acid was rinsed off and the veneers were dried. Next, the silane (Porcelain Primer, Bis Silane A&B, Bisco, USA) was brushed to the intaglio surface of the etched veneers and was air-dried after 30 s.

At the same time, the prepared surface of each tooth was etched with 37% phosphoric acid (Total Etch, Ultradent, South Jordan, UT, USA) for 15 s. The acid was thoroughly rinsed off for 20 s and the tooth was air-dried. Next, the bonding agent (All-Bond Universal, BISCO Inc, Schaumburg, IL, USA) was applied to the surface, gently dried and light cured for 10 s.

The final step was applying a very thin layer of porcelain bonding resin (Porcelain Bonding Resin, Bisco Inc, Schaumburg, IL, USA) and then the resin cement (Choice 2, BISCO Inc., Schaumburg, IL, USA) to the intaglio surface of laminates. They were seated with finger pressure on the corresponding prepared teeth. After curing for 5 s the excess cement was removed, and all the surfaces were light cured for 40 s.

All the veneer preparations and cementation steps had been performed by the same operator.

### 2.4. Thermocycling and Cyclic Loading

For artificial aging, the cemented laminates were 5000 thermocycled between 5 and 55°C with 30 s immersion time at each temperature using water as a medium.

**Figure 3:**
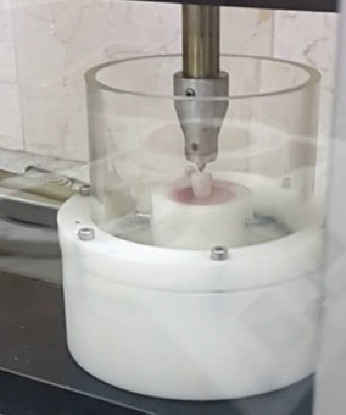
Cyclic loading.

Then, by using a surveyor, each specimen was vertically embedded up to CEJ in the resin-filled sample holder mold of the chewing simulator machine (CS4, SD Mechatronic GmbH, Westerham, Germany). After setting of the resin, the incisal edge of each specimen was vertically loaded 500,000 cycles (30 N at 4 Hz) (Figure 3). By completion of cyclic loading, all the specimens were evaluated for cracks or fractures under a stereo microscope (Olympus Bx 60; Olympus Optical) at ×12.5 magnification. None of them showed any signs of cracks or chipping.

### 2.5. Measuring Fracture Resistance

**Figure 4:**
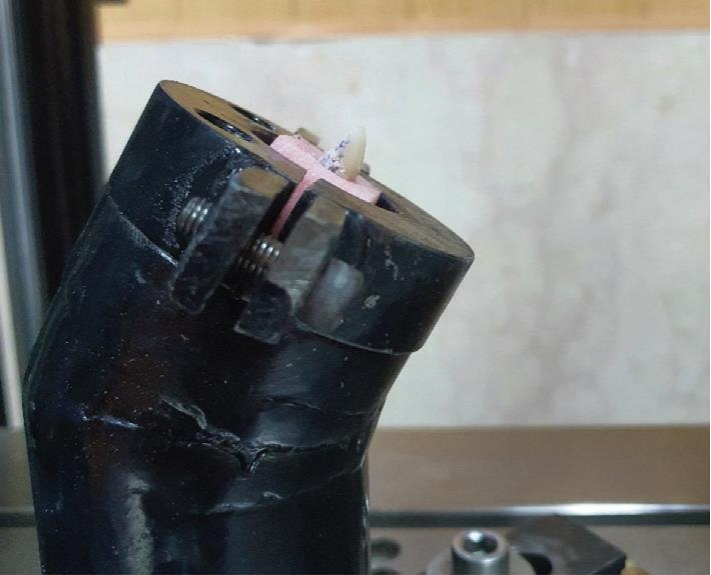
Fracture resistance test.

In order to measure fracture resistance, each specimen was fixed on a 135° angled metal holder (Figure 4) and transferred to a universal testing machine (Model 5585H; Instron Corp, Norwood, Mass). A 3.5 mm round tip rod was positioned on the incisal edge of each laminate veneer and was loaded at a crosshead speed of 1 mm/min until catastrophic failure occurred. The failure load was recorded in newtons (N).

### 2.6. Mode of Failure

**Figure 5:**
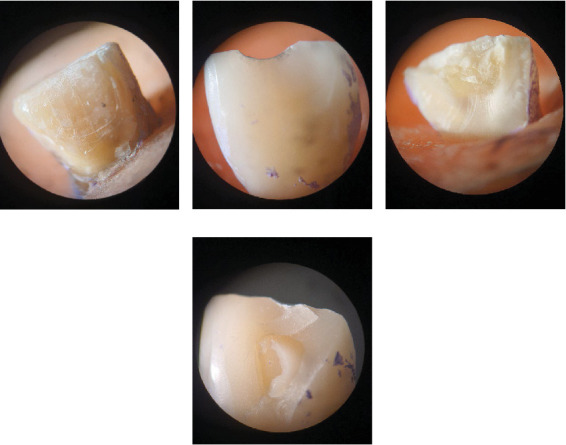
Mode of Failure in specimens. (a) Adhesive failure, (b) ceramic cohesive failure, (c) tooth cohesive failure, and (d) mixed failure.

All restorations were inspected under a stereomicroscope (×12.5) magnification for the mode of the catastrophic failure according to the following classification: (1) ceramic cohesive failure: fracture only in ceramic; (2) adhesive failure: fracture only in resin cement; (3) cohesive tooth failure: fracture only in tooth structure; (4) mixed failure: fracture in both tooth structure and ceramic. (Figure 5a–d)

### 2.7. Statistical Analysis

Parametric statistical analyses were performed at a 95% confidence interval (CI) using statistical software (SPSS, IBM Corp., Version 22.0). The data were analyzed using a two-way analysis of variance (ANOVA), with incisal reduction height and type of material as the variables, followed by *T*-test to evaluate differences among the groups.

## 3. Results

The mean load to failure, minimum, maximum and standard deviation of fracture resistance of veneers are presented in Table 1. The results of two- way ANOVA test showed that the interaction between ceramic material and laminate incisal height was significant (*p* value = 0.048). In LD veneers, increasing ceramic height significantly increased load to failure (*p*=0.025). However, in PICN veneers, the difference between groups was not significant (*p*=0.94) (Table 2).

The highest mean load to failure value was in group LD3.5 (900 N) and the lowest in PICN 3.5 groups (460 N). This value was not significantly different between LD2 (620 N) and PICN 2 (466 N).

The mode of failure of different groups is summarized in Table 3. In PICN groups, the predominant mode was cohesive ceramic fracture (62.5%) that is repairable. They had no adhesive failure between veneer and the tooth.

Group LD3.5 had the least fracture of ceramic (25%), the most root fracture (37.5%) that is irreparable failure, and the debonding of veneers (12.5%).

The mode of failure in LD2 group was closer to PICN gropus.

## 4. Discussion

The goal of this study was to evaluate the effect of incisal height on the maximum load to fracture of e.max CAD and VITA Enamic laminate veneers. Since incisal height only significantly affected the load to failure of LD veneers, the null hypothesis was partially rejected.

The results of this study are very close to what is reported by Chen et al. [28], although they have compared fracture resistance of e.max CAD and LAVA Ultimate (a resin nanoceramic) at different thicknesses (0.5, 1.0, 1.5, 2.0, 3.0 mm). In their study, the load to failure of sandblasted LAVA Ultimate did not change significantly with the change of thickness. On the contrary, the fracture load of e.max CAD did not change too much from 0.5 to 1.5 mm but significantly increased at 2 and 3 mm [28]. They explained the contrast by the different stiffness of composite resins and ceramics. In resins, unlike stiffer ceramics, the stress concentrates in the loading point, and when the accumulated stress becomes higher than the material's strength, cracks occur, penetrate deeper and lead to final fracture. On the other hand, since Young's modulus of e.max CAD is high, it becomes stiffer when its thickness increases, and as a result, it can withstand higher loads. Another similarity between the two studies is that at 2 mm thickness, e.max CAD and sandblasted LAVA Ultimate had almost the same load to fracture, as in the present study that the fracture resistance of VITA Enamic and e.max CAD laminate veneers at 2 mm incisal height was not significantly different [28].

Andrade et al. [29] investigated the effect of thickness (0.6 and 1.5 mm) on the fracture resistance of ceramic occlusal veneers made of VITA Enamic, IPS e.max CAD, and LAVA Ultimate. They reported that at 0.6 mm the fracture resistance of materials was not significantly different, but e.max CAD had significantly higher resistance at 1.5 mm. Again, VITA Enamic did not show significant increase in fracture resistance by increasing thickness [29]. Czechawski et al. [30] made similar study on IPS Empress Esthetic, VITA Enamic, IPS e.max Press, and Ceramill Zolid occlusal veneers at 1, 1.5, and 2 mm thicknesses. In their study the fracture resistance of VITA Enamic and e.max CAD was not different at 1 mm but significantly different at 2 mm. The results of both studies are in agree with the present study, although they aimed for occlusal rather than labial veneers [29, 30]. In addition, Taha et al. [31] assessed the effect of two margin designs and the occlusal thicknesses (2 mm and 3.5 mm) on the fracture resistance of endodontically treated teeth restored with VITA Enamic endocrown restorations. The results showed that although endocrowns with 3.5 mm occlusal thickness had higher mean fracture resistance values, but the difference between the two thicknesses was not statistically significant. In all three studies, like the present study, the fracture resistance of VITA Enamic was not influenced by thickness [29–31].

On the other hand, Akoğlu and Gemalmaz [22] and Schmidt et al. [17] studied the effect of preparation design and incisal reduction (2 and 4 mm) on the fracture resistance of laminates made of IPS Empress I (leucite-reinforced pressable ceramic) and reported reduction of the load to failure at 4 mm incisal height. Nevertheless, Ge et al. [8, 32] evaluated the effect of porcelain and enamel thickness on the failure load of laminate veneers and concluded that increasing feldspathic porcelain thickness from 0.2 to 1.4 mm slightly decreased the load needed to form initial cone cracks but markedly increased the load needed to produce terminal catastrophic fracture if the preparation is only in enamel. Schweiger et al. [33] studied fracture load as a function of the thickness of IPS Empress CAD, IPS e.max CAD, and LAVA Plus. They reported an increase in the fracture load of IPS Empress CAD when the thickness increased, although it was not as much as two other materials. It is not clear whether the kind of manufacturing of IPS Empress restoration (pressing or milling) or the difference in the methodology of the studies is the reason for the dissimilarity between results. Akoğlu and Gemalmaz [22] and Schmidt et al. [17] had studied laminates bonded to natural teeth that are closer to clinical situations, and Schweiger et al. [33] made their research on discs.

Even so, the reports of decreased fracture resistance of IPS Empress I (leucite-reinforced ceramic) with increased thickness cannot be extended to lithium disilicate IPS e.max CAD because the latter is stronger and has higher fracture toughness. According to Apel et al. [34] although there is a microbridging strengthening mechanism in leucite-based glass ceramics, the crack propagates through both the crystals and glass phase. On the other hand, in lithium disilicate glass ceramics, the crack only propagates through the glass matrix without affecting high strength crystals [34]. Bakeman et al. [35] in their study on posterior partial coverage restorations, reported that the fracture load of IPS e.max press was significantly higher than IPS Empress I, and increasing the thickness of ceramics increased the load to fracture, although the difference was not significant. The superior strength of IPS e.max CAD in comparison with IPS Empress I is confirmed by other studies as well [36, 37].

In the literature there are reports of decreased flexural strength and fracture resistance in bilayered ceramics when the thickness of the layering porcelain was increased from 1 to 3 mm. The result was ascribed to the higher residual tensile stress in the thicker porcelain [38, 39].

The common mode of failure in PICN groups was cohesive ceramic fracture (62.5%) that might be attributed to their lower load to failure (460 N). The least ceramic fracture was in the LD3.5 group (25%) that had the highest load to failure (900 N). Only in the LD3.5 group there was adhesive failure in cement and debonding of laminates (12.5%). It might be due to the high strength of both ceramic and tooth and that the weakest link had been the bond between cement and ceramic/tooth in. On the other hand, the LD3.5 group had the highest cohesive fracture of the tooth near the CEJ or at the root. In these specimens, the laminates remained bonded to the tooth without damage, and the load was transferred to the other parts of the tooth. Although the mean load to failure of PICN groups was the same, there were some instances of higher values in PICN2 group. This may explain why there was cohesive tooth fracture (12.5%) in this group but not in the PICN3.5 laminates. The failures that only include ceramic or debonding of laminates are repairable. Any fracture of a tooth is irreparable. The catastrophic failure of IPS e.max CAD restorations is reported in other research [36, 40–42].

In this study, the palatal chamfer design was selected for the incisal edge preparation because it increases bonding surface area and prevents crack propagation by providing a rounded angle [43].

Since in clinical condition the temperature and water exposure have negative effects on the glass phase of ceramics, thermal cycling was performed in the present study [44]. To duplicate the oral condition and normal function inside the mouth, the specimens were 500,000 cyclic loaded, which is equivalent to 2 years of average mastication [29]. The specimens were vertically loaded at incisal edges because in other directions the loading rod of the chewing machine would slide. However, in the fracture resistance test the specimens were loaded at 135° to the palatal surface since the average angulation between upper and lower incisors is 135° [11]. Despite having different incisal heights, in all groups, 1 mm from the incisal edge and on the palatal side of the laminates was selected for applying static load. Further distance would result in the crosshead tip of the universal testing machine to be either on the ceramic-tooth junction or on the tooth structure.

The mean force to failure, in all groups of the present study, was higher than the reported mean bite force values of 150 N in the anterior area [45], suggesting that both materials can be indicated for laminate veneers regardless of the thickness.

There are limitations for the current study. First of all, only two materials were examined, so the results cannot be extended to other ceramics. Second, although in this in vitro study it was attempted to duplicate oral conditions by carrying out thermal and cyclic loading tests, it certainly cannot be complete, and the results must be tested inside the mouth (in vivo). Third, the study was performed on human central incisors in order to have the strength, thermal conductivity, elasticity, and bonding properties of natural teeth and better duplication of the clinical condition. However, the teeth varied in age and quality, making the bonded interface difficult to standardize among the specimens.

## 5. Conclusion

According to the results of this study, in VITA Enamic laminates, increasing the incisal height has no effect on the fracture load. However, increasing the incisal height of IPS e.max CAD laminates increases the fracture resistance. Therefore, in loss of incisal height situations, this material might be suggested for laminate veneer restorations.

## Figures and Tables

**Table 1:** The mean load to failure values of specimens (N).

Fracture resistance of ceramic	Minimum	Maximum	Mean	Standard deviation
LD				
2.00	382.59	896.11	620.1138	213.49070
3.50	652.17	1202.62	901.8163	233.32564
PICN				
2.00	211.33	788.28	466.4338	221.76025
3.50	222.00	569.62	460.1363	108.44397

**Table 2:** *t*-Test for equality of means.

Ceramic			Levene's test for equality of variances		*t*-Test for equality of means						
			*F*	Sig.	*t*	df	Sig. (2-tailed)	Mean difference	Std. error difference	95% confidence interval of the difference	
										Lower	Upper
LD	Fracture resistance	Equal variances assumed	0.658	0.431	−2.519	14	0.025	−281.70250	111.81409	−521.51988	−41.88512
		Equal variances not assumed	—	—	−2.519	13.891	0.025	−281.70250	111.81409	−521.69665	−41.70835

PICN	Fracture resistance	Equal variances assumed	6.964	0.019	0.072	14	0.943	6.29750	87.27665	−180.89229	193.48729
		Equal variances not assumed	—	—	0.072	10.167	0.944	6.29750	87.27665	−187.73540	200.33040

**Table 3:** Mode of failure of specimens.

Ceramics	Cohesive ceramic fracture (%)	Adhesive fracture (%)	Cohesive tooth fracture (root fracture) (%)	Mixed fracture (%)
LD 2 mm	50	0	12.50	37.50
LD 3.5 mm	25	12.50	37.50	25
PICN 2 mm	62.50	0	12.50	25
PICN 3.5 mm	62.50	0	0	37.50

## Data Availability

The data that support the findings of this study are available from the corresponding author upon reasonable request.
